# Amelioration of aging-induced muscular decline by black soybean (*Rhynchosia nulubilis*) and black rice (*Oryza sativa* L.) extracts

**DOI:** 10.3389/fimmu.2025.1554941

**Published:** 2025-03-19

**Authors:** Hyejeong Park, Seungmin Yu, Wooki Kim

**Affiliations:** ^1^ Department of Food and Nutrition, Yonsei University, Seoul, Republic of Korea; ^2^ Precision Nutrition Research Group, Korea Food Research Institute (KFRI), Wanju, Republic of Korea

**Keywords:** diet, energy metabolism, mitochondria, phytochemical, sarcopenia

## Abstract

Aging leads to a decline in the mass and function of skeletal muscles, a condition known as sarcopenia. It was previously reported that aging-related alterations in protein degradation, chronic inflammation, and deterioration of mitochondrial metabolism affect the acceleration of muscle atrophy in the elderly. However, the detailed mechanism or substantial causes for age-related muscle loss are still lacking, yet exercise or an increment in dietary protein intake are suggested as effective approaches to mitigate muscle atrophy. This study aims to investigate the regulatory effect of black soybean (*Rhynchosia nulubilis*) and black rice (*Oryza sativa* L.) mixture extract (BBME), which are rich in protein and bioactive compounds, in 12-month-old aged mice and L6 myotubes. BBME was orally administered at 300 and 600 mg/kg/day (low and high doses) for 12 weeks, and its effects on systemic glucose homeostasis and skeletal muscle metabolism were evaluated. Consequently, BBME at a high dose marginally ameliorated muscle loss and significantly improved glucose metabolism. BBME also reduced cellular senescence markers and enhanced mitochondrial biogenesis in aged skeletal muscles. Additionally, BBME exerted insulin-like activity by promoting glucose metabolism in L6 myotubes. These findings suggest the potential of BBME as a functional food ingredient in alleviating aging-induced muscle loss by modulating mitochondrial activity and glucose metabolism.

## 1 Introduction

Skeletal muscle is the largest organ that comprises about 40% of body weight. It is well known that skeletal muscle is involved in physical performance, breathing, thermogenesis, and posture (1). Besides, skeletal muscle contributes to basal energy metabolism and serves as a reservoir for ingested nutrients while providing fuels for other tissues (2). Moreover, skeletal muscle is the main site where many different metabolic processes occur including glucose metabolism and glycogen synthesis (3). In particular, muscle tissues have been regarded as modulators of glucose homeostasis. In healthy individuals, approximately 80% of extracellular glucose is utilized by skeletal muscle under the postprandial state. Under insulin or contraction-stimulated conditions, glucose transporter proteins, including the most abundant glucose transporter 4 (GLUT4), carry glucose molecules into muscle cells, playing a pivotal role in glucose homeostasis (4, 5).

Skeletal muscles are exhibited to sensitively respond to environmental changes through their structural and functional plasticity (6). In this regard, a well-balanced diet with appropriate physical exercise leads to muscle hypertrophy, yet malnutrition and aging may result in muscle atrophy with an increased risk of metabolic syndrome (7). Accordingly, aging-induced loss of muscular function and mass in the elderly severely causes a decrement in longevity and healthy life span. Several studies have suggested mitochondrial dysfunction, increment of protein degradation, and chronic inflammation as the main factors in the decline of muscle function, but detailed mechanisms are still unknown (8, 9). Since age-related changes may affect the function and structure of skeletal muscle, energy metabolism and synthesis are altered in muscle tissues (8, 10). Furthermore, the deterioration of glucose metabolism with aging impairs skeletal muscle and exacerbates the aforementioned factors (11). Thus, the maintenance of glucose homeostasis and related metabolism might be a key factor in achieving successful aging with the inhibition of muscular loss (12).

Black soybean (*Rhynchosia nulubilis*) is widely used as a dietary protein source and is also rich in bioactive compounds such as anthocyanins, isoflavones, and saponins (13). Likewise, black rice (*Oryza sativa* L.) contains not only essential nutrients including carbohydrates and proteins but also beneficial components like anthocyanins, vitamins, phytosterols, and phenolic compounds (14). Several studies have reported anti-inflammatory, anti-obesity, anti-diabetic, and anti-cancer properties of anthocyanins and isoflavones extracted from black soybean and black rice (15–17). In addition to bioactive compounds, black soybean and black rice are known for their high protein content, which is crucial for muscle growth and hypertrophy (18). Furthermore, increasing protein intake may lead to suppressed muscle loss by promoting skeletal muscle protein synthesis in the elderly (19–21). Although numerous studies have been conducted to investigate the health-promoting effects of anthocyanins, isoflavones, and phytosterols extracted from black soybean and black rice, few studies have investigated their effects on muscle function or glucose metabolism (22).

Chronic, low-grade inflammatory milieu with aging has been indicated as a major risk factor for a decline in muscle mass and function (23). In this regard, a previous study has shown an anti-inflammatory effect of black soybean and black rice mixture extract (BBME) in elderly mice (24). Therefore, this study aimed to determine whether BBME supplementation could mitigate aging-induced muscle decline by modulating cellular senescence, inflammation, and mitochondrial function. The dietary effect of BBME on aged skeletal muscle function was investigated, and its underlying mechanism was examined by evaluating glucose uptake and utilization in L6 myotubes.

## 2 Materials and methods

### 2.1 Black soybean and black rice mixture extract preparation

Black soybean and black rice extracts were prepared according to the procedures previously described (24). Briefly, black soybean (Bibong herb, Yangju, Korea) and black rice (Bibong herb) were mixed at the ratio of 4:1 (w:w) and extracted in 30% ethanol at 60°C for 6 h. Black soybean and black rice mixture extract was then filtered and concentrated. The black soybean and black rice mixture extract was stored at -20°C until further use.

### 2.2 Animal study

12-month-old elderly male C57BL/6 mice were purchased from Korea Basic Science Institute (KBSI, Gwangju, Korea) and syngeneic young male mice at 4 weeks after birth were obtained from Raon Bio (Yongin, Korea). Mice were housed at 20°C in a 12 h light/dark cycle. AIN-76A rodent diet and water were provided *ad libitum* during the experimental period. Aged mice were randomly divided into three groups (Aged control, BBME-L, and BBME-H) after 1 week of acclimation. Elderly mice in BBME-L group were orally gavaged with BBME of 300 mg/kg/day, while BBME-H group received 600 mg/kg/day BBME for 12 weeks (n = 6). Young and aged control mice were orally gavaged with tap water during the dietary intervention. The body weight and food intake of mice were monitored twice a week. One week before sacrifice, an oral glucose tolerance test (OGTT) was performed by gavaging 2 g/kg of glucose following overnight fasting. The blood glucose concentration of tail veins was measured by a glucometer (Accu-Check Performa, Roche Diagnostics GmbH, Mannheim, Germany) at 0 (before oral gavage), 30, 60, 90, 120 min after glucose treatment and the area under the curve was calculated (25). Following OGTT, mice were fed appropriate diets for another week. At the end of the dietary intervention, mice were euthanized via CO_2_ inhalation. Blood samples were collected by cardiac puncture and stored at 4°C for 3 h. Then, blood sera were obtained by centrifugation at 300 ×g for 5 min. Blood glucose levels in blood sera were measured with a clinical analyzer (Hitachi, Tokyo, Japan). Gastrocnemius, tibialis anterior, extensor digitorum longus, and soleus tissues were collected and stored at – 80°C for further experiments.

### 2.3 Cell culture and BBME treatment

Rat L6 myoblasts were purchased from Korean Cell Line Bank (KCLB, Seoul, Korea). L6 myoblasts were grown in Dulbecco’s modified Eagle’s medium (DMEM, Welgene, Gyeongsan, Korea) containing 10% fetal bovine serum (FBS, Welgene) and 1% antibiotic/antimycotic solution (10,000 U/mL penicillin G, 10,000 μg/mL streptomycin, and 25 μg/mL amphotericin B, Welgene). Cells were then seeded in 24-well plate for differentiation into myotubes by culturing in DMEM with 2% horse serum (HS, Thermo Fisher Scientific, Waltham, MA, USA) for 5 days. The differentiation medium was changed every 2 days. Differentiated cells were serum-starved for 4 h in serum-free medium. Then, serum-starved cells were treated with BBME for 3 h or insulin for 1 h before further experiments.

### 2.4 Glucose uptake assay

Glucose uptake was assessed using 2-(N-(7-Nitrobenz-2-oxa-1,3-diazol-4-yl)Amino)-2-Deoxyglucose (2-NBDG, Thermo Fisher Scientific) solution diluted in Krebs-Ringer-Phosphate-HEPES (KRPH) buffer (Bio-solution, Suwon, Korea) following BBME or insulin treatment. Cells were washed twice with phosphate-buffered saline (PBS, Welgene) and incubated with 100 μM of 2-NBDG solution at 37°C for 30 min. Residual glucose was washed with pre-chilled PBS and cells were detached with Trypsin-EDTA (Thermo Fisher Scientific). Then, cells were resuspended in PBS for flow cytometry analysis. Glucose uptake was determined by measuring mean fluorescence intensity (MFI) at FL1 channel of a BD Accuri C6 flow cytometer (BD Biosciences, San Jose, CA, USA).

### 2.5 Measurements of glycolytic rate and mitochondrial respiration

Energy metabolism such as glycolysis and oxidative phosphorylation in differentiated myotubes was evaluated by an extracellular flux analyzer (Seahorse XF, Agilent Technologies, Palo Alto, CA, USA). L6 myoblasts were seeded in XF cell culture plate and differentiated into myotubes. L6 myotubes were then serum-starved and further treated with BBME or insulin. For measuring glycolysis in differentiated myotubes, glycolytic rate assay was performed. The oxygen consumption rate (OCR) and extracellular acidification rate (ECAR) were assessed by injecting 5 μM rotenone & antimycin A (Rot/AA) and 500 mM 2-dexoyglucose (2-DG) sequentially. The glycolytic proton efflux rate (glycoPER), the rate of protons extruded during glycolysis, was determined by using OCR and ECAR. Basal glycolysis (last glycoPER before rotenone/antimycin A injection) and compensatory glycolysis (maximum glycoPER after rotenone/antimycin A injection) of myotubes were calculated following measurement (26). To evaluate mitochondrial oxidative phosphorylation, real-time OCR was assessed under basal condition and after treatment of oligomycin, carbonyl cyanide-4 (trifluoromethoxy)phenylhydrazone (FCCP), and rotenone/antimycin A. The mitochondrial respiration parameters, basal respiration, maximal respiration, ATP production, and spare respiratory capacity, were calculated by following equations:


$$\begin{array}{c} \text{Basal respiration}=\left(\text{Last OCR before oligomycin injection}\right)\\ -\left(\text{Non-mitochondrial Respiration Rate}\right) \end{array}$$

$$\begin{array}{c} \text{Maximal respiration}=\left(\text{Maximum OCR after FCCP injection}\right)\\ -\left(\text{Non-mitochondrial Respiration Rate}\right) \end{array}$$

$$\begin{array}{c} \text{ATP production}=\left(\text{Last OCR before oligomycin injection}\right)\\ -\left(\text{Minimum OCR after oligomycin injection}\right) \end{array}$$

$$\begin{array}{c} \text{Spare respiratory capacity}=\left(\text{Maximal respiration}\right)\\ -\left(\text{Basal respiration}\right) \end{array}$$

Following energy metabolism measurements, protein contents of the cells were quantified by bicinchoninic acid (BCA) assay (Abcam, Cambridge, UK) for normalization of the metabolic profiles of differentiated myotubes to the protein content per well as an indicator of cell counts.

### 2.6 RNA extraction and qRT-PCR

BBME-treated and insulin-stimulated myotubes were harvested to assess the transcription of glucose transporter 4 (GLUT4). Total RNA was isolated from harvested cells with HiGene Total RNA Prep Kit (Biofact, Daejeon, Korea) following the manufacturer’s instructions. Collected hindlimb muscles were used to analyze the effect of BBME on aging-related changes in muscle function. Total RNA was extracted by homogenizing tissues with Tri-reagent (Favorgen, Taiwan). The purity and concentration of RNA were measured with Nanodrop One Microvolume UV-Vis Spectrophotometer (Thermo Fisher Scientific). Quantitative reverse transcriptase PCR (qRT-PCR) was conducted using 2X One step qRT-PCR Master Mix (SYBR Green, Biofact) and CFX Connect™ Real-Time PCR detection system (Bio-Rad, Hercules, CA, USA) under the 2 step conditions: 50°C for 30 min, 95°C for 15 min, and 40 cycles of 95°C for 20 s and 60°C for 40 s. The sequences of primers used for qRT-PCR are listed in **Table 1**. Relative mRNA quantification was determined using the delta-delta Ct method (2^–ΔΔCt^). The delta Ct value was calculated by subtracting Ct value of the reference gene (GAPDH or β-actin) from Ct value of target gene. Then, the delta delta Ct value was determined by evaluating difference between average Ct value of control group and Ct value of experimental group.

**Table 1:** Primer sequence.

Gene	Primer sequence
*glut4 (Slc2a4)*_Rat	Forward: 5′-GGCTGTGCCATCTTGATGAC-3′ Reverse: 5′-CACGATGGACACATAACTCATGGA-3′
*GAPDH*_Rat	Forward: 5′-ATGACTCTACCCACGGCAAG-3′ Reverse: 5′-CTGGAAGATGGTGATGGGTT-3′
*p53*	Forward, 5′-CCGACCTATCCTTACCATCATC-3′ Reverse, 5′-TTCTTCTGTACGGCGGTCTC-3′
*p16*	Forward, 5′-CCCAACGCCCCGAACT-3′ Reverse, 5′-GCAGAAGAGCTGCTACGTGAA-3′
*il6*	Forward, 5′-CAAAGCCAGAGTCCTTCAGA-3′ Reverse, 5′-TTGGTCCTTAGCCACTCCTT-3′
*tnfα*	Forward, 5′-AAATGGCCTCCCTCTCATCAG-3′ Reverse, 5′-GTCACTCGAATTTTGAGAAGATGATC-3′
*hexokinase II*	Forward, 5′-GGAACCCAGCTGTTTGACCA-3′ Reverse, 5′-CAGGGGAACGAGAAGGTGAAA-3′
*pyruvate kinase*	Forward, 5′-TTGACTCTGCCCCCATCAC-3′ Reverse, 5′-GCAGGCCCAATGGTACAAAT-3′
*glut4*_Mouse	Forward, 5′-ATGGCTGTCGCTGGTTTCTC-3′ Reverse, 5′-ACCCATGCCGACAATGAAGT-3′
*pgc1α*	Forward, 5′-TTCCACCAAGAGCAAGTAT-3′ Reverse, 5′-CGCTGTCCCATGAGGTATT-3′
*β-actin*_Mouse	Forward, 5′-AGGCCCAGAGCAAGAGAG-3′ Reverse, 5′-GGGTGTTGAAGGTCTCAAAC-3′
*GAPDH*_Mouse	Forward, 5′-AACACTGAGCATCTCCCTCA-3′ Reverse, 5′-GTGGGTGCAGCGAACTTTAT-3′

### 2.7 Statistical analysis

All data are expressed as mean ± standard error of the mean (SEM). Statistical significance of results was analyzed with one-way analysis of variance (ANOVA) and *post-hoc* Tukey’s multiple comparison test using Prism 9 software (GraphPad Software, La Jolla, CA, USA). p < 0.05 was considered as statistically significant. Significant differences between experimental groups were indicated as different alphabets.

### 2.8 Ethics statement

All procedures and experiments were confirmed by the Institutional Animal Care and Use Committee (IACUC) of Kyung Hee University (Approval ID: KHGASP-21-057).

## 3 Results

### 3.1 Marginal increment of skeletal muscles in aged mice by BBME

As aging-associated body weight gain increases the risk of metabolic diseases including obesity and diabetes (27, 28), the effects of BBME administration on body weight and muscle mass in aged mice were initially assessed. Following oral intervention for 12 weeks, there was no significant difference in body weight change among aged mice (**Supplementary Figure 1A**). Since aging involves the loss of muscle functioning, the mass of skeletal muscles was measured and normalized to whole body weight. As expected, elderly mice exhibited a significantly decreased ratio of muscle mass/whole body weight (7.94 ± 0.36 mg/g) as compared to that of young mice (11.52 ± 0.50 mg/g, p < 0.05), indicating aging diminishes the mass of muscles in mice. In these elderly mice, BBME exhibited an increasing tendency of muscle mass (BBME-L 8.39 ± 0.40, and BBME-H 8.90 ± 0.55 mg/g) (p > 0.05, **Supplementary Figure 1B**).

### 3.2 Amelioration of impaired glucose tolerance in aged mice by BBME

**Figure 1:**
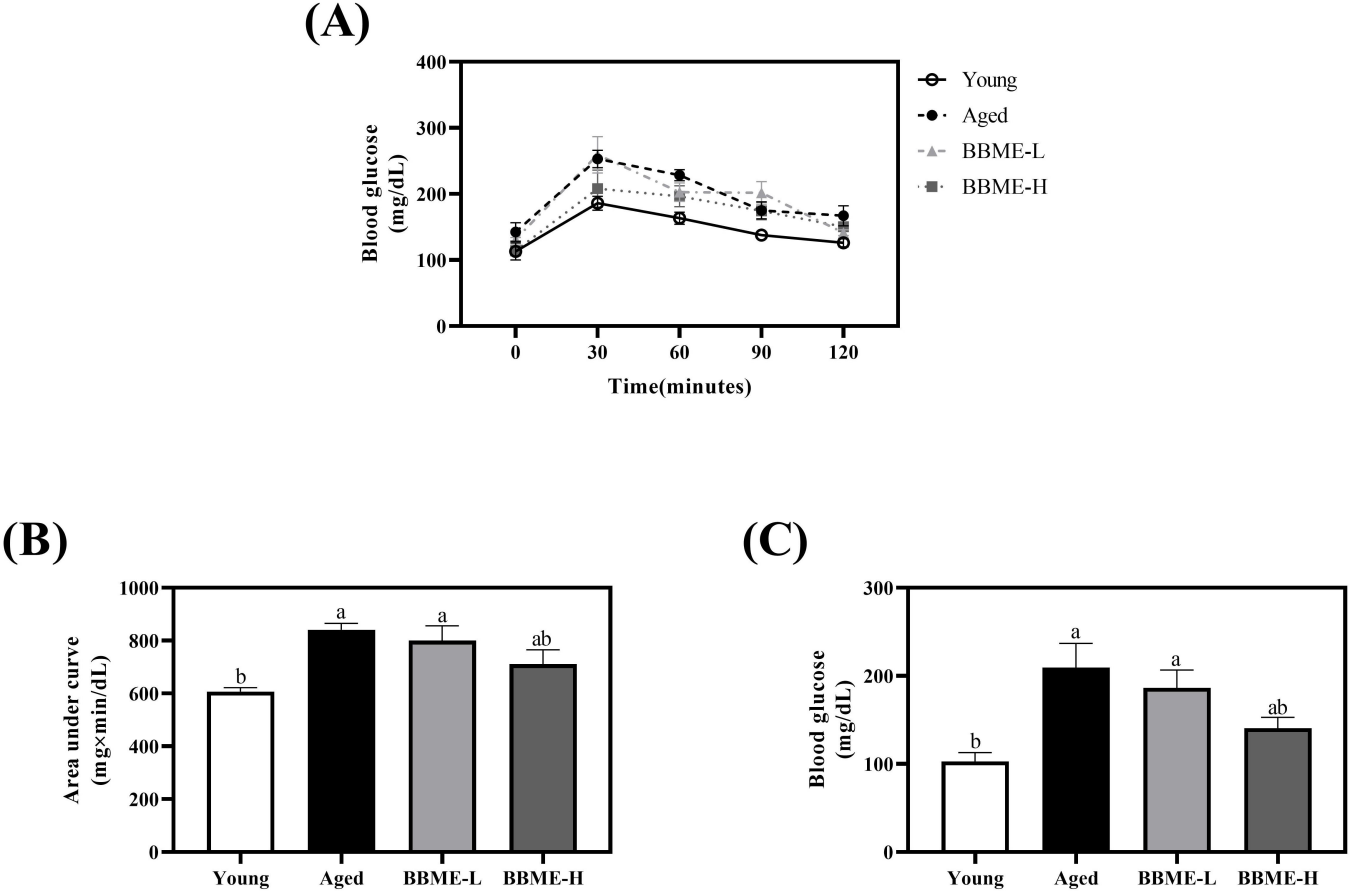
Oral glucose tolerance test (OGTT) result and fasting blood glucose level of aged mice. During the oral administration of BBME, OGTT was performed in young and aged mice **(A)**. Area under the curve (AUC) was calculated **(B)**. After the sacrifice of mice, serum blood glucose levels were measured **(C)**. Data are presented as mean ± SEM (n = 5). Significant differences are expressed with different letters (p < 0.05) [BBME-L, low dose of BBME (300 mg/kg/day); BBME-H, high dose of BBME (600 mg/kg/day)].

Aging also increases the risk of diabetes, which highlights the importance of appropriate glucose metabolism for the prevention of geriatric diseases (29, 30). Therefore, the current study sought the effect of BBME on glucose metabolism, as determined by an oral glucose tolerance test. Elderly control and BBME-L-fed mice exhibited a steep increase of blood glucose concentration after oral gavage, while BBME at the higher dose (BBME-H) revealed a comparable slope to that of Young control (**Figure 1A**). The area under the curve (AUC) also significantly increased in Aged group (841.50 ± 23.55 mg×min/dL) and BBME-L group (800.40 ± 55.52 mg×min/dL) compared to Young group (606.90 ± 15.49 mg×min/dL) (p < 0.05, **Figure 1B**). Of interest, a high concentration of BBME (BBME-H: 711.50 ± 54.31 mg×min/dL) attenuated increment of AUC without any statistical difference to Aged control group (p > 0.05). Besides OGTT which assesses the short-term utilization of glucose following oral administration, fasting serum glucose concentration in a physiological status was further measured after the sacrifice. Aging significantly increased serum glucose concentration, which may reflect the deterioration of glucose homeostasis. As shown in **Figure 1C**, the glucose concentration of Aged (209.40 ± 27.40 mg/dL) and BBME-L (186.40 ± 20.14 mg/dL) mice were significantly higher than that of young mice (102.80 ± 10.06 mg/dL, p < 0.05). In comparison, a high dose of BBME (BBME-H) to elderly mice lowered serum glucose (140.60 ± 12.21 mg/dL) statistically comparable to young mice. Taken together, BBME at a high dose marginally improved glucose homeostasis in aged mice, potentially reducing the risk of age-related metabolic diseases.

### 3.3 Suppression of aging-induced muscular senescence and inflammation by BBME

**Figure 2:**
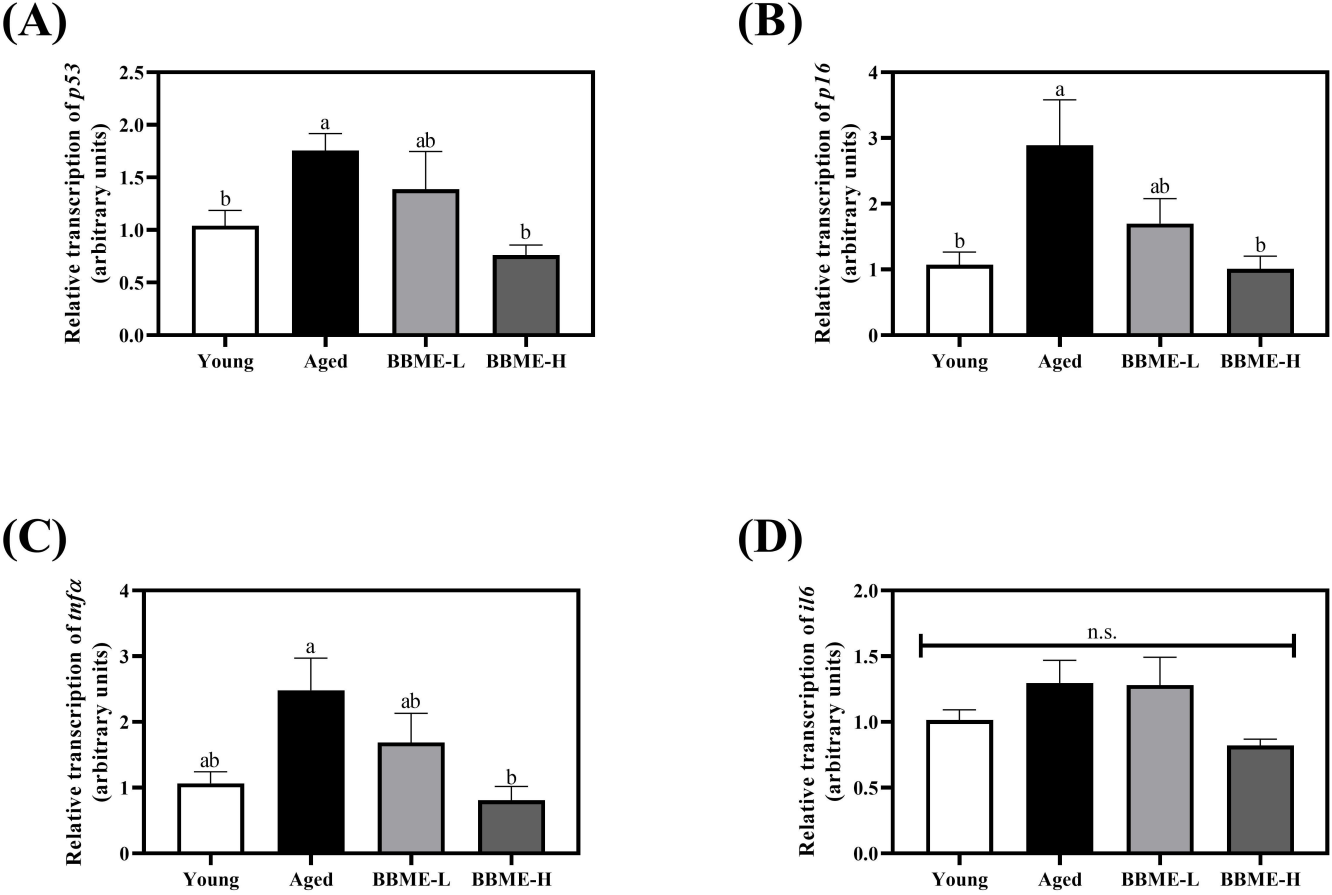
Senescence-associated secretory phenotype (SASP) gene transcription in skeletal muscles. Skeletal muscle tissues were dissected and the transcription of SASP factors **(A)** *p53*, **(B)** *p16*, **(C)** *tnfα*, and **(D)** *il6* was assessed by qRT-PCR. Relative transcript levels of genes were calculated using the delta-delta Ct method (2^−ΔΔCt^). Values are presented as mean ± SEM (n = 5) and different letters indicate statistically significant differences (p < 0.05, n.s., no significant difference) [BBME-L, low dose of BBME (300 mg/kg/day); BBME-H, high dose of BBME (600 mg/kg/day)].

Since BBME exerted a tendency to improve glucose homeostasis with aging, the dietary effect of BBME on the decline in muscle function was further examined. Diverse stimuli up-regulate p53 and p16 pathways in muscle cells during aging and induce cellular senescence. Cells then exhibit the senescence-associated secretory phenotype (SASP), as characterized by the production of inflammatory cytokines, immune modulators, growth factors, and proteases, following activation of NF-κB signaling pathway (31, 32). In this regard, the transcription levels of cellular senescence and inflammation-related indicators were measured by qRT-PCR in muscle tissue lysates following dietary intervention of BBME in elderly mice. Aging-induced up-regulation of *p53* transcription was suppressed with oral gavage of BBME, especially at a higher dose (BBME-H) with comparable transcription to that of Young control (p < 0.05, **Figure 2A**). A similar tendency was observed in the transcription of *p16*, for which a high dose of BBME (BBME-H) also significantly down-regulated *p16* mRNA transcription compared to Young group (p < 0.05, **Figure 2B**). In addition, oral administration of BBME also attenuated muscle inflammation by decreasing *tnfα* mRNA transcription (p < 0.05, **Figure 2C**), a hallmark of SASP, although BBME did not significantly affect transcription of *il6* in aged mice (p > 0.05, **Figure 2D**). These findings suggest that a potent of BBME in mitigating cellular senescence and inflammation, thereby improving muscle function during aging.

### 3.4 Modulation of glucose metabolism and mitochondrial biogenesis by BBME in skeletal muscle

**Figure 3:**
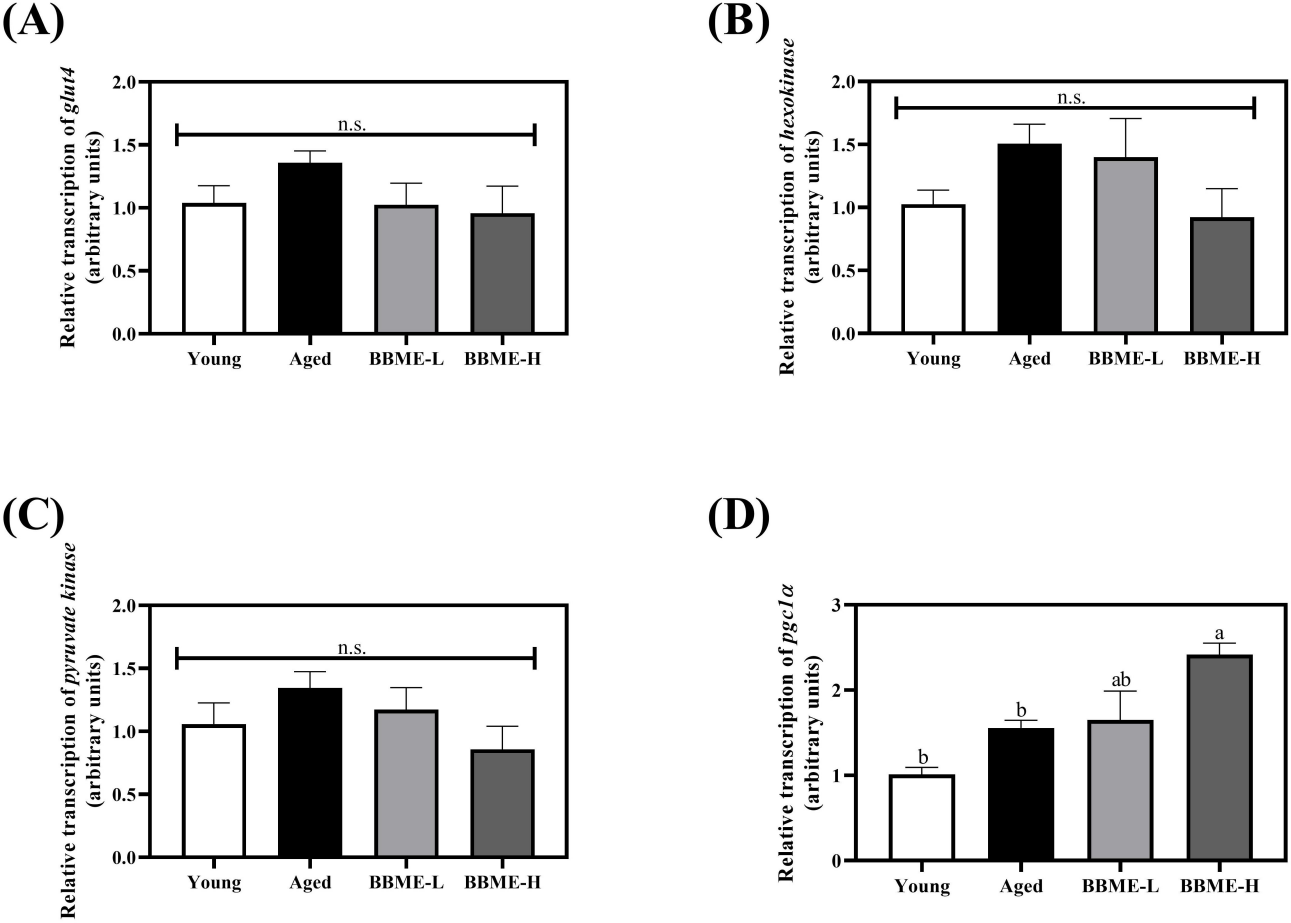
Relative transcript levels of glycolytic enzymes and mitochondrial regulator in aged muscles. Transcription of glucose metabolism-related genes **(A)** *glucose transporter 4* (*glut4*), **(B)** *hexokinase*, and **(C)** *pyruvate kinase* was evaluated. The mRNA transcription level of mitochondrial regulator, *pgc1α* was assessed by qRT-PCR **(D)**. The relative gene expression was calculated with delta-delta Ct method (2^−ΔΔCt^). Data are expressed as mean ± SEM (n = 5). Significant differences were indicated with different letters (p < 0.05, n.s., no significant difference) [BBME-L, low dose of BBME (300 mg/kg/day); BBME-H, high dose of BBME (600 mg/kg/day)].

It was reported that aging-induced obesity, as well as high fat feeding to middle-aged rats, decreases the expression of GLUT4 in skeletal muscles by shortening its mRNA poly(A) tails (33, 34). To elucidate metabolic changes, the transcription of glucose transporter *glut4*, glycolytic *hexokinase*, and *pyruvate kinase* was quantified by qRT-PCR following BBME administration (**Figures 3A–C**). In aged mice, an increasing tendency was observed, but BBME did not exhibit any statistical change (p > 0.05) in the mRNA transcription, indicating that BBME mitigates inflammaging-induced muscle loss and SASP independently of glucose metabolism. Furthermore, as aged muscle is characterized by impaired mitochondrial function and biogenesis, the transcription level of peroxisome proliferator-activated receptor-gamma coactivator 1-alpha (*pgc1α*), a regulator of mitochondrial function, was assessed (**Figure 3D**). At higher dose, BBME significantly up-regulated transcription of *pgc1α* as compared to Aged mice (p < 0.05). While BBME did not affect glucose metabolism in aged muscles, an increment of *pgc1α* indicates BBME may enhance mitochondrial function and energy metabolism in aged muscles, which is critical for maintaining muscle health.

### 3.5 Promoted glucose transport and utilization in L6 myotubes by BBME

**Figure 4:**
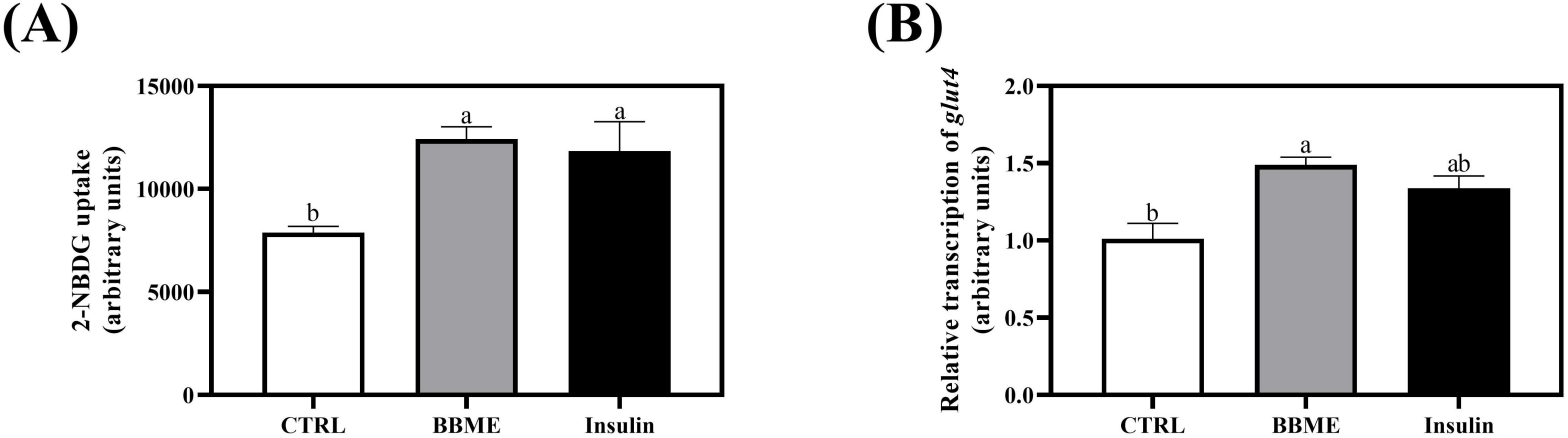
Glucose transport and uptake in differentiated L6 myotubes. Following incubation with 100 nM of insulin and 100 ppm of BBME, **(A)** 2-NBDG uptake of myotubes and **(B)** transcription of *glucose transporter 4* (*glut4*) were assessed by flow cytometry and qRT-PCR, respectively. Values are expressed as mean ± SEM (n = 3). Different letters show statistically significant differences (p < 0.05, n.s., no significant difference) (CTRL, non-treated control group).

As evidenced in OGTT and fasting blood glucose concentration, BBME aids in the lowering of blood glucose. Therefore, the effect of BBME on the glucose transport and utilization in skeletal muscle cells was further studied in L6 myotubes at 100 ppm determined by preliminary cell cytotoxicity assay (**Supplementary Figure 2**). The cellular glucose uptake was determined by measuring the mean fluorescence intensity (MFI) of transported 2-NBDG. BBME significantly increased glucose uptake to 12,414.43 ± 606.59 MFI compared to non-treated control (7,885.31 ± 298.59 MFI), exhibiting a similar increment with insulin-stimulated cells (11,844.20 ± 1,421.30 MFI) (p < 0.05, **Figure 4A**). Similarly, mRNA transcription of *glut4* was also significantly up-regulated following BBME treatment (p < 0.05, **Figure 4B**).

**Figure 5:**
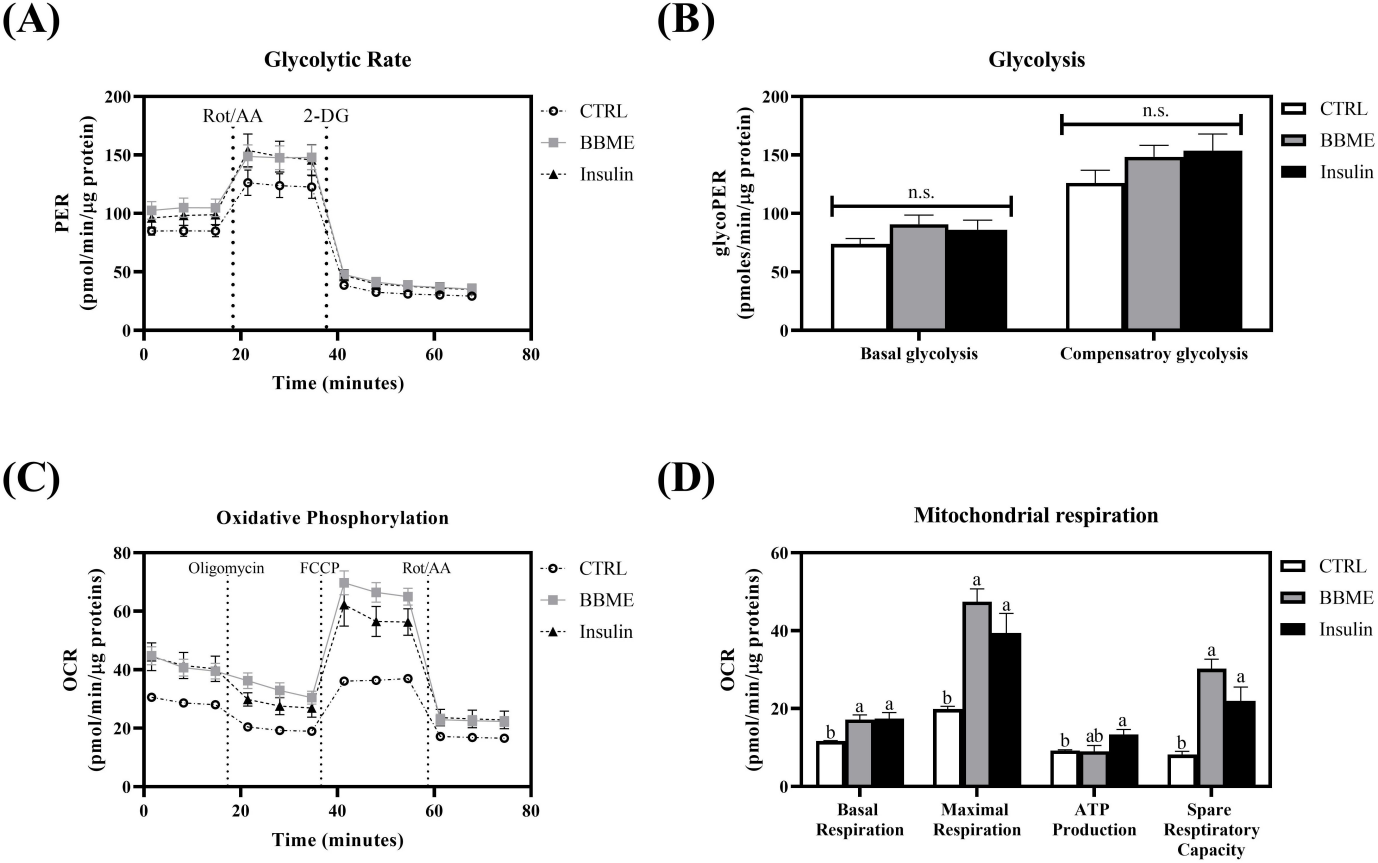
Metabolic profiles in L6 myotubes. **(A)** The glycolytic proton efflux rate of myotubes was evaluated by Seahorse extracellular flux analyzer with sequential injection of rotenone & antimycin A and 2-DG (2-deoxyglucose). **(B)** Basal glycolysis and compensatory glycolysis were calculated and normalized with protein concentration. **(C)** The real-time oxygen consumption rate of differentiated myotubes was measured by Seahorse extracellular flux analyzer treating oligomycin, FCCP, and rotenone & antimycin A. **(D)** Basal respiration, maximal respiration, ATP production, and spare respiratory capacity were calculated and normalized with protein concentration. Data are presented as mean ± SEM (n = 3). Significant differences are indicated with different letters (p < 0.05, n.s.: no significant difference) (CTRL, non-treated control group).

Considering that BBME-treated cells showed a similar tendency in glucose uptake with insulin-stimulated myotubes, further assessments of glycolysis and mitochondrial oxidative phosphorylation (OXPHOS) were conducted to determine whether increased glucose uptake would lead to the activation of energy metabolism. The glycolytic parameters were first measured in differentiated myotubes. The slight increases were observed in basal and compensatory glycolysis in BBME and insulin-treated cells without any statistical significance (p > 0.05, **Figures 5A, B**) (Basal glycolysis; CTRL: 73.88 ± 4.66, BBME: 90.79 ± 7.76, and Insulin: 86.06 ± 8.22 pmoles/min/μg protein) (Compensatory glycolysis; CTRL: 126.07 ± 10.79, BBME: 148.36 ± 9.90, and Insulin: 153.76 ± 14.08 pmoles/min/μg protein). Even though BBME significantly up-regulated cellular uptake of glucose, its oxidation by glycolysis was marginally increased.

Acetyl-CoA, the cytoplasmic metabolite of glycolysis, further enters into mitochondrial respiration for the production of ATP (35, 36). In order to assess the effect of BBME on specific phases of mitochondrial energy metabolism, OCR of myotubes was analyzed following sequential injection of oligomycin, FCCP, and rotenone & antimycin A. As shown in **Figure 5C**, oxidative phosphorylation was increased with the treatment of BBME compared to non-treated control group (CTRL). Specifically, BBME and insulin significantly increased basal respiration (BBME: 17.20 ± 1.17 and Insulin: 17.43 ± 1.53) within experimental group (CTRL: 11.67 ± 0.11 pmoles/min/μg protein). In parallel, BBME significantly elevated maximal respiration (47.43 ± 3.30) compared to non-treated control group (17.70 ± 1.46) exhibiting insulin-like activity (Insulin: 39.40 ± 5.01 pmoles/min/μg protein). Accordingly, ATP production was increased in both BBME and insulin-treated groups (CTRL: 9.23 ± 0.21, BBME: 9.95 ± 0.60, and Insulin: 13.34 ± 1.30 pmoles/min/μg protein). Spare respiratory capacity of BBME and insulin-treated cells were also up-regulated (BBME: 30.23 ± 2.46 and Insulin: 21.97 ± 3.53) exhibiting significant difference with non-treated control group (8.19 ± 0.82 pmoles/min/μg protein) (p < 0.05, **Figure 5D**). These results demonstrate that BBME not only enhances glucose uptake but also improves mitochondrial function in muscle cells, indicating its potential as a dietary supplement for improving metabolic health and muscle function in aging populations.

## 4 Discussion

Skeletal muscle is known to participate not only in body movement but also in energy metabolism, thereby supporting the functioning of other tissues (2, 37). However, muscle function deteriorates naturally with aging, leading to a decline in muscle mass, strength, and overall functionality (38). Furthermore, increased inflammation, impaired glucose metabolism, and the development of metabolic diseases are also known to exacerbate muscle degeneration during aging (39, 40). While exercise, increased protein, and nutrient intake, and the targeting of key metabolic pathways have been proposed to mitigate muscle loss, the detailed mechanisms underlying aging-related muscle atrophy remain unclear (41–44).

Black soybean (*Rhynchosia nulubilis*) and black rice (*Oryza sativa* L.) are both rich in bioactive compounds such as anthocyanins, isoflavones, and saponins (45, 46). These components have been shown to exert anti-inflammatory, antioxidant, anti-cancer, and anti-diabetic effects, positioning black soybean and black rice as valuable dietary components for promoting overall health (47, 48). In addition to preclinical findings, several studies have demonstrated the health benefits of black rice and black soybean in human subjects. Black soybean has inhibited fact accumulation and improved vascular function in overweight and obese individuals (49–51). Similarly, black rice has reduced obesity-related parameters and enhanced cognitive function in postmenopausal and elderly populations (52, 53). Given their high protein content and essential amino acids, this study assessed the dietary effect of BBME extract on aged mice focusing on aging-induced muscle loss (45, 54).

The body weight change and muscle mass were first measured. Although BBME administration did not significantly alter body weight, there was a marginal increase in muscle mass. In this regard, several studies previously demonstrated enhancement of muscle function and alleviation of muscle atrophy yet no changes of muscle mass were observed (55–57).

Furthermore, BBME significantly improved glucose metabolism in aged mice, as evidenced by the attenuation of glucose spikes during OGTT and a reduction in fasting blood glucose levels. This finding is consistent with previous reports that black soybean extract and anthocyanins from black rice modulated glucose metabolism in diabetic and obese mice, respectively (58, 59). These results suggest that BBME has the potential to mitigate age-related glucose metabolism deterioration, though further research is needed to fully understand the underlying mechanisms.

Since BBME, particularly at a high dose, significantly modulated glucose homeostasis in aged mice, further investigation using harvested skeletal muscles was conducted by measuring several factors that induce muscle loss. For cellular senescence and inflammatory gene transcription, aging increased the transcription of *p53*, *p16*, and *tnfα* compared to Young control, the *il6* mRNA was slightly increased without statistical significance. BBME administration, however, significantly reversed aging and inflammatory gene transcription consistent with a previous study in which regulation of systemic inflammation was suppressed by BBME in aged mice (24).

Given that BBME can ameliorate inflammatory state in skeletal muscles of elderly mice, the underlying mechanisms of metabolic changes in muscles were further studied by assessment of mRNA transcription in glucose metabolism and mitochondrial functioning. Aging-induced muscle hypotrophy has also been shown to cause malfunctioning of glucose metabolism and mitochondrial respiration accompanied by a decrement of glycolytic enzymes and metabolites (60). In addition, to assess metabolic changes in aged skeletal muscles, the mRNA levels of glucose transporters and glycolytic enzymes were evaluated. BBME did not affect the transcription of glucose transporter or glycolytic enzymes, however, BBME significantly increased *pgc1α* mRNA level in a dose-dependent manner compared to Aged control. Given the role of PGC-1α in systemic inflammation and glucose metabolism, the increment of its transcription implies that BBME mitigated aging-related degenerations by reducing inflammatory signals potential in skeletal muscles (61, 62). BBME mitigated skeletal muscle decline by enhancing systemic glucose homeostasis, reducing inflammation, and promoting mitochondrial biogenesis. Several studies have reported that black soybean, black rice, and their derivatives promote muscle function by activating the AMPK pathway and PGC-1α (63, 64). Similarly, the beneficial effects of BBME may also be attributed to the activation of these pathways.

The main function of skeletal muscle is to maintain glucose homeostasis, with glucose serving as the primary fuel for muscle contraction (65). Along with the regulatory effect of BBME in maintaining glucose homeostasis and mitigating aging-related changes observed in aged mice, further investigations were conducted using L6 myotubes to elucidate the role of BBME in modulating glucose metabolism and mitochondrial function. The glucose uptake and transcription of glucose transporter were initially measured in differentiated L6 myotubes. Consequently, BBME markedly stimulated glucose uptake through up-regulation of *glut4* transcript level in skeletal muscle. Consistent with previous studies that reported the regulatory effect of anthocyanins from black bean and black rice in inducing glucose utilization in muscle cells, the mixture of black soybean and black rice similarly increased glucose uptake in L6 myotubes (66, 67).

Following glucose uptake, muscle cells mediate mitochondrial metabolism by up-regulating ATP synthesis through promoted glucose oxidation and oxidative phosphorylation (68). Therefore, glycolysis and oxidative phosphorylation in L6 myotubes were assessed following the treatment of BBME. A slight increase in basal and compensatory glycolysis was observed in BBME- or insulin-treated myotubes, although there were no significant differences among all experiment groups. However, BBME significantly increased basal respiration, maximal respiration, and spare respiratory capacity, similar to insulin-stimulated control, indicating its potential to enhance mitochondrial function. Taken together, insulin-stimulated glucose uptake and further improved mitochondrial function by increasing oxidative phosphorylation as previously reported (68, 69). Of interest, BBME also up-regulated glucose uptake and oxidative phosphorylation comparable to insulin, indicating its potential in regulating glucose metabolism and mitochondrial respiration.

Consistent with the increased *pgc1α* transcript level observed in BBME-treated aged mice, BBME enhanced mitochondrial function in L6 myotubes. Since PGC-1α activation is closely linked to AMPK and SIRT1 pathways, BBME may enhance muscle function by stimulating these metabolic regulators, thereby improving glucose uptake and mitochondrial respiration (70, 71). The significant increase in GLUT4 expression in L6 myotubes further supports this hypothesis, suggesting a role for AMPK activation in BBME-mediated glucose homeostasis. This aligns with previous reports demonstrating that black soybean, black rice and their bioactive compounds promote skeletal muscle function by promoting glucose uptake and utilization (58, 67, 72). These findings suggest that BBME may protect against muscle dysfunction by promoting mitochondrial biogenesis *in vivo* and enhancing mitochondrial activity *in vitro*.

Overall, these results indicate that BBME has the potential to modulate muscle loss and functional decline associated with aging by improving glucose metabolism, reducing inflammaging, and enhancing mitochondrial function. To confirm the potential of BBME as a dietary intervention for age-related muscle deterioration, further studies are required to elucidate the mechanism underlying the protective effect of BBME on age-induced changes. Moreover, as several studies have demonstrated the dietary effect of anthocyanins or plant proteins on muscle function, further clinical studies are needed to validate the potential benefits of BBME on human muscle function, metabolic health, and sarcopenia.

## 5 Conclusion

The current study investigated the beneficial effect of dietary BBME on muscle function in aged mice. As reported, aging can lead to weight gain, muscle loss, and impaired glucose metabolism, which were partially reversed by BBME administration. However, the oral administration of BBME reversed those alterations in aged mice. Specifically, a high dose of BBME exerted a modulatory effect on glucose homeostasis and potentially ameliorated senescent phenotype skeletal muscle, suggesting that BBME has potential for alleviating age-related changes. BBME also significantly stimulated glucose uptake and utilization of glucose by promoting oxidative phosphorylation in L6 myotubes. Interestingly, BBME did not significantly affect glucose metabolism in aged skeletal muscle while stimulating glucose uptake and utilization *in vitro*. The discrepancy may result from the lowered absorption and distribution of active compounds in animals as compared to the direct, higher treatment of the extracts to cells. Therefore, to confirm these observations, additional studies on protein synthesis or muscular structure in aged mice as well as the molecular mechanism underlying increased glucose metabolism, are needed to fully elucidate the dietary effect of BBME. Thus, further human clinical trials are needed to evaluate the efficacy, optimal dosage, and safety of BBME in the elderly to confirm these findings in human subjects. Given that aging-induced changes in muscle, especially loss of weight and function, may lead to sarcopenia, this study indicates that protein- and anthocyanin-rich black soybean and black rice could be potential regulators for alleviating muscle loss caused by aging.

## Data Availability

The original contributions presented in the study are included in the article/**Supplementary Material**. Further inquiries can be directed to the corresponding author.
