# Supplementary material for: Amelioration of aging-induced muscular decline by black soybean (Rhynchosia nulubilis) and black rice (Oryza sativa L.) extracts
Source: Front Immunol. 2025 Mar 19;16:1554941. doi: 10.3389/fimmu.2025.1554941 (PMC11961972; doi:10.3389/fimmu.2025.1554941)
Supplement: Supplementary file 1 [file DataSheet1.docx]

**Supplementary Figures**

**
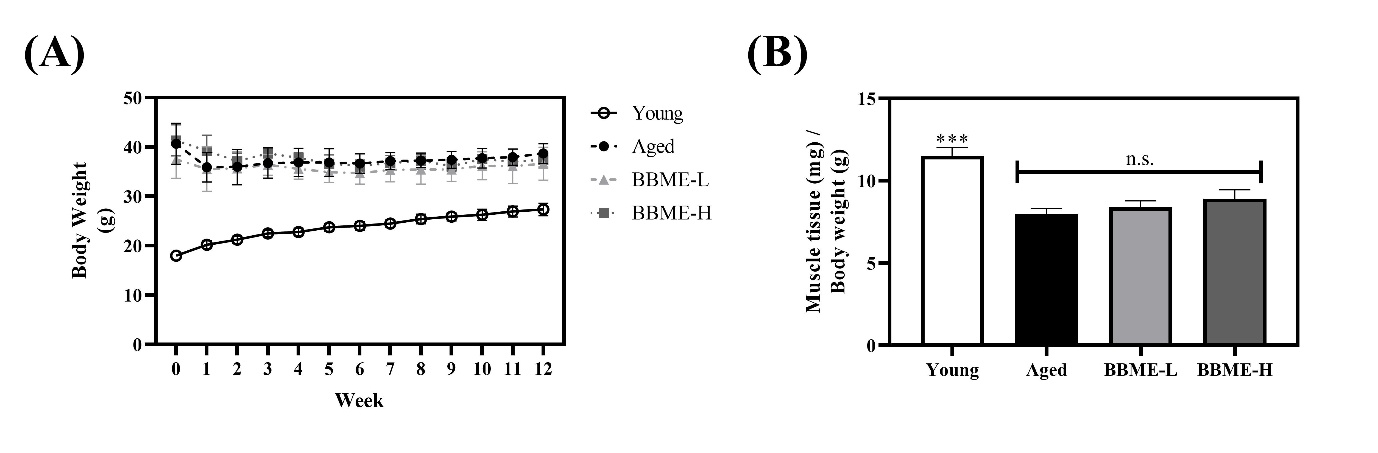
**

**Supplementary Figure 1. Body weight and muscle weight changes in mice**

(**A**) Mouse body weight changes were evaluated twice a week. (**B**) The muscle weight of each group was measured and expressed as the ratio of muscle weight to the final body weight. Data are indicated as mean ± SEM (n = 6). P-values were obtained using Student’s *t*-tests. Asterisks represent a significant difference with Aged group (***p < 0.001, n.s.: no significant difference) (BBME-L, low dose of BBME (300 mg/kg/day); BBME-H, high dose of BBME (600 mg/kg/day)).


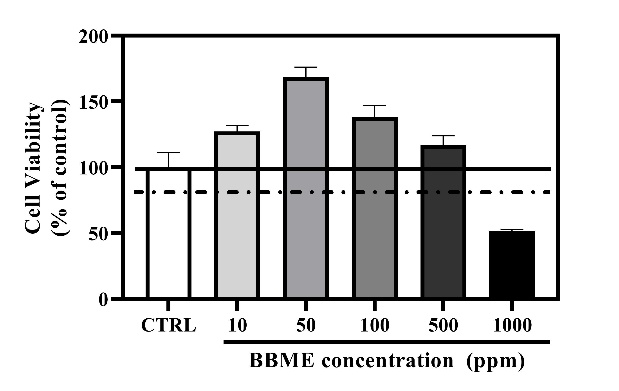


**Supplementary Figure 2. Cell cytotoxicity test following BBME treatment in L6 myotubes**

The cell viability of L6 myotubes was determined by MTT assay. BBME was treated at the concentrations of 10, 50, 100, 500, and 1000 ppm for 24 h. MTT solution was added and the cell viability was analyzed by measuring absorbance at 540 nm. Data are indicated as mean ± SEM (n = 4) (CTRL, non-treated control group).
